# Effects of nitrogen concentration on growth, fucoxanthin production, and fatty acid composition of the marine diatom *Nitzschia* sp

**DOI:** 10.3389/fpls.2026.1936445

**Published:** 2026-10-14

**Authors:** David Kwame Amenorfenyo, Wenquan Zheng, Junhao Huang, Ke Peng, Hui Guo, Qianru Li, Feng Li, Yuewen Deng

**Affiliations:** 1 College of Fisheries, Guangdong Ocean University, Zhanjiang, China; 2 Guangdong Provincial Key Laboratory of Aquatic Animal Disease Control and Healthy Culture, Zhanjiang, China

**Keywords:** docosahexaenoic acid (DHA), eicosapentaenoic acid (EPA), fucoxanthin, nitrogen concentration, *Nitzschia* sp., PUFA

## Abstract

**Background:**

Nitrogen availability strongly regulates growth and biochemical accumulation in marine diatoms, but the magnitude and direction of these responses are strain-specific. Optimizing nitrate supply may therefore provide a practical approach for improving biomass production and the accumulation of high-value metabolites in diatoms.

**Methods:**

The effects of four sodium nitrate (NaNO₃) concentrations (0, 75, 150, and 300 mg L^−1^) on growth, biomass accumulation, total carotenoid concentration, fucoxanthin production, and fatty acid composition were investigated in the marine diatom *Nitzschia* sp. The standard f/2 nitrate concentration (75 mg L^−1^ NaNO₃) was used as the reference treatment, while 0, 150, and 300 mg L^−1^ represented nitrogen starvation, two-fold nitrate enrichment, and four-fold nitrate enrichment, respectively. Growth, biomass, carotenoid, and fucoxanthin parameters were monitored during 12 days of cultivation, and fatty acid composition was determined at the end of the cultivation period.

**Results:**

Nitrate availability strongly affected growth and biochemical accumulation. The highest cell density (2.7 × 10⁶ cells mL^−1^ on day 9) and biomass productivity (0.068 ± 0.027 g L^−1^ d^−1^ on day 6) were obtained under 300 mg L^−1^ NaNO₃. Total carotenoid concentration reached its maximum of 2.08 mg L^−1^ on day 9 under 150 mg L^−1^ NaNO₃. Fucoxanthin content reached its highest value of 8.42 mg g^−1^ DW under 300 mg L^−1^ NaNO₃ on day 3, accompanied by the highest fucoxanthin productivity (0.689 ± 0.175 mg L^−1^ d^−1^). After 12 days, the highest total identified fatty acid content was obtained under 150 mg L^−1^ NaNO₃ (179.39 mg g^−1^ DW). EPA and DHA yields also reached their maximum values under 150 mg L^−1^ NaNO₃, at 25.12 and 3.52 mg g^−1^ DW, respectively.

**Conclusion:**

Nitrate concentration differentially affected biomass production and biochemical accumulation in Nitzschia sp. A high nitrate supply of 300 mg L^−1^ NaNO₃ favored biomass accumulation and early fucoxanthin production, whereas 150 mg L^−1^ NaNO₃ favored total identified fatty acid accumulation and EPA/DHA yields. These findings demonstrate that nitrate supply should be optimized according to the target product and provide strain-specific baseline information for further optimization of high-value compound production in *Nitzschia* sp.

## 1 Introduction

Microalgae are diverse photosynthetic microorganisms with rapid growth rates and the capacity to synthesize proteins, lipids, vitamins, minerals, and photosynthetic pigments (Benoiston et al., 2017; Wang et al., 2018; Sathasivam et al., 2019; Dolganyuk et al., 2020; Gao et al., 2024). They are the primary biological producers of many marine omega-3 fatty acids and are therefore being explored as sustainable alternatives to fish oil for aquafeed and nutraceutical applications (Soto-Sánchez et al., 2023; Siddiqui et al., 2024; Balasubramaniam et al., 2025). Among them, diatoms are ecologically important primary producers that contribute substantially to marine carbon fixation, nutrient cycling, and food-web functioning (Benoiston et al., 2017). Their high photosynthetic efficiency is associated with a specialized light-harvesting apparatus containing chlorophyll a, chlorophyll c, and fucoxanthin–chlorophyll protein complexes (Nagao et al., 2020). In addition to their ecological importance, diatoms have received increasing attention as potential cell factories for the production of high-value pigments and nutritionally valuable fatty acids (Büchel, 2020).

Fucoxanthin is a xanthophyll carotenoid predominantly found in brown algae, diatoms, haptophytes, and other chromophyte algae. It serves as an accessory light-harvesting pigment and contributes to photoprotection under fluctuating light conditions (Pajot et al., 2022).​ Fucoxanthin has attracted considerable scientific and commercial interest because of its reported antioxidant, anti-inflammatory, anticancer, antidiabetic, and hypolipidemic activities, most of which have been demonstrated primarily *in vitro* and in preclinical studies. These bioactivities support its potential use in food, nutraceutical, pharmaceutical, and cosmetic applications (Méresse et al., 2020; Xiao et al., 2020; Lourenço-Lopes et al., 2021; Mohibbullah et al., 2022; Remya et al., 2022; Jawaji et al., 2023; Ghallab et al., 2024; Zhang et al., 2024). Diatoms are particularly promising sources of fucoxanthin because this carotenoid is a major light-harvesting pigment and an integral component of their photosynthetic apparatus (Bai et al., 2022).

Diatoms are also recognized as important producers of long-chain polyunsaturated fatty acids (PUFAs), particularly eicosapentaenoic acid (EPA; C20:5n3) and docosahexaenoic acid (DHA; C22:6n3).​ These omega-3 PUFAs are essential nutrients for both humans and aquatic animals. Because the endogenous conversion of shorter-chain fatty acids to EPA and DHA is limited in humans and many animals, dietary intake of these fatty acids remains important (Shahidi and Ambigaipalan, 2018).

Nitrogen is one of the major nutrients that regulates diatom growth and metabolism because it is required for the synthesis of amino acids, proteins, nucleic acids, chlorophylls, and enzymes involved in photosynthesis and carbon assimilation (Yaakob et al., 2021). Changes in nitrogen availability can alter cell division, photosynthetic activity, pigment biosynthesis, carbon allocation, and lipid metabolism. Under nitrogen limitation, microalgae commonly show reduced growth and altered photosynthetic pigment composition typically through a decline in chlorophyll a and, in many diatoms, in fucoxanthin, whereas other carotenoids may be retained or increase, while reallocating carbon toward storage compounds, however, the magnitude and direction of these responses are strongly species- and strain-dependent (Levitan et al., 2015; Remmers et al., 2018; Truong et al., 2024). Transcriptomic and metabolomic studies in the model diatom *Phaeodactylum tricornutum* have further shown that nitrogen stress induces extensive remodeling of central carbon metabolism, nitrogen recycling, lipid metabolism, and photosynthetic processes (Levitan et al., 2015; Remmers et al., 2018). These findings demonstrate that nitrogen availability affects not only biomass formation but also the quality and composition of commercially relevant metabolites.

The effects of nitrate supply on fucoxanthin and fatty acid production have been investigated in several diatoms and other microalgae. For example, nitrate availability influences biomass production, lipid accumulation, EPA content, fucoxanthin concentration, and chrysolaminarin production in *P. tricornutum* cultivated in a photobioreactor (Gao et al., 2017). In marine diatoms, nitrogen and iron availability have also been reported to modify growth, fucoxanthin and lipid content, and fatty-acid profiles (Sahin et al., 2019). Similarly, different nitrate concentrations affected the co-production of fucoxanthin and fatty acids in *Conticribra weissflogii* (Rui et al., 2023). In *Nitzschia* sp. NW129, nitrogen concentration influenced both fucoxanthin accumulation and lipid production, confirming the potential of nitrate management as a practical strategy for diatom biorefinery processes (Cao et al., 2022).

Nitrogen responses are modulated, beyond species and strain identity, by nitrogen source and concentration, culture stage, irradiance, temperature, salinity, silicon and phosphorus availability, and cultivation system (Kumar and Bera, 2020; Yaakob et al., 2021; Grubišić et al., 2024; Nath et al., 2026), and therefore cannot be generalized across diatoms. The genus *Nitzschia* includes marine, brackish-water, and freshwater taxa with considerable physiological diversity (Mucko et al., 2021). Several *Nitzschia* strains have been identified as potential producers of fucoxanthin and EPA (Cao et al., 2022; Khaw et al., 2022; Lu et al., 2023), however, information remains limited for many isolates, particularly regarding the simultaneous effects of nitrogen supply on biomass, carotenoids, fucoxanthin, and EPA/DHA accumulation.

In the present study, sodium nitrate (NaNO_3_) was used as the nitrate source because nitrate is the nitrogen form conventionally supplied in f/2 medium and is widely used for laboratory cultivation of marine diatoms. The 75 mg L^-1^ NaNO_3_ treatment corresponded to the nitrate concentration in standard f/2 medium and was therefore selected as the reference condition. The 0, 150, and 300 mg L^-1^ NaNO_3_ treatments represented nitrogen starvation, two-fold nitrate enrichment, and four-fold nitrate enrichment, respectively. These concentrations corresponded to approximately 0, 0.88, 1.76, and 3.53 mM NaNO_3_. They were selected to establish a controlled laboratory nutrient gradient rather than to reproduce a single natural nitrate regime. Although nitrate concentrations in coastal waters can vary widely with freshwater input, upwelling, mixing, and anthropogenic nutrient loading, the enriched treatments used here were higher than nitrate concentrations generally found in oligotrophic open-ocean waters. Thus, the experiment was designed to assess the physiological and biotechnological responses of this isolate under nitrogen-starved and nitrate-enriched culture conditions.

Accordingly, this study investigated the effects of four NaNO_3_ concentrations (0, 75, 150, and 300 mg L^-1^) on growth, biomass accumulation, total carotenoid concentration, fucoxanthin production, and fatty-acid composition in a marine *Nitzschia* sp. isolate. The objective was to identify nitrate conditions that could support biomass formation while improving the accumulation of high-value compounds, particularly fucoxanthin, EPA, and DHA.

## 2 Materials and methods

### 2.1 Algal strain isolation and identification

*Nitzschia* sp. was isolated from the coastal waters of Changsha Village, Xiangshan County, Zhejiang Province, China (water temperature 22 °C, salinity 25, pH 8 at the time of sampling) and purified by the Laboratory of Algae Resources Development and Breeding Environment Ecological Restoration (Guangdong Ocean University). Taxonomic identification was performed based on morphological observations under an Olympus BX53 fluorescence microscope. However, 18S rDNA sequencing was not conducted in this study. Therefore, the isolate is reported as *Nitzschia* sp.

### 2.2 Culture conditions

*Nitzschia* sp. cells were cultured in sterilized f/2 medium (seawater-based) at 25.0 ± 2 °C under continuous illumination intensity of 30 ± 2 μmol photons m^-2^ s^-1^. Cells used for experiments were harvested during the exponential growth phase by centrifugation at approximately 1,800 × g for 8 min and resuspended in fresh medium prior to inoculation.

### 2.3 Experimental design

The nitrate experiment was conducted using seawater-based f/2 medium modified with four NaNO_3_ concentrations: 0, 75, 150, and 300 mg L^-1^. The 75 mg L^-1^ NaNO_3_ treatment corresponds to the nitrate concentration of standard f/2 medium. Therefore, the four treatments represented a nitrogen-starved, standard (f/2 nitrate supply), two-fold nitrate enrichment, and four-fold nitrate enrichment, respectively.

Cultures were grown for 12 days in 2,000 mL borosilicate Erlenmeyer flasks containing 700 mL culture volume. The initial cell density was approximately 1.1 × 10^6^ cells mL^-1^. Cultures were maintained at the temperature and irradiance described in Section 2.2, at salinity 25 and with continuous aeration. Each treatment was performed in biological triplicate. Samples were collected every three days for cell density, biomass, pigment, and fucoxanthin analyses. Fatty acid composition was determined at the end of the 12-day cultivation period.

### 2.4 Cell density, biomass concentration, and productivity

Cell density was determined using a hemocytometer under an Olympus BX53 microscope. Lugol’s iodine solution was added to fix the cells before counting.

(1) $$DW\;\left(g/L\right)\;=\;\left(M_{2}-\;M_{1}\right)\;\times \;10^{3}/V$$

Dry weight (DW) was determined gravimetrically. Briefly, 10 mL of algal culture was filtered through pre-weighed and pre-dried mixed cellulose ester membranes (47 mm diameter, 1 μm nominal pore size; Shanghai Xingya Purification Material Factory, Shanghai, China). Filters were washed with deionized water to remove residual salts, dried at 80 °C overnight, cooled in a desiccator, and weighed using an analytical balance with 0.1 mg precision. Dry weight was calculated according Equation 1:

where M_1_ is the pre-dried filter weight (g), M_2_ is the post-drying filter + biomass weight (g), and V is the filtration volume (mL).

Biomass productivity was calculated using Equation 2 as follows:

(2) $$BP\;\left(gL^{-1}d^{-1}\right)\;=\;DW_{t}-DW_{0}/t$$

where DW*_t_* and DW*_0_* are biomass concentrations at time (t) and day 0, respectively.

### 2.5 Carotenoid and fucoxanthin extraction and analysis

Total carotenoids were determined using the spectrophotometric method by Jeffrey and Humphrey (1975). Briefly, 10 mL of algal culture was centrifuged at approximately 1,000 × g for 3 min. Pigments were extracted with 90% acetone in the dark at 4 °C until the biomass was colorless. Extracts were centrifuged again at approximately 1,000 × g for 3 min, and absorbance was measured using a UV–visible spectrophotometer at 480, 510, 630, 664, and 750 nm using 90% acetone as the blank. Total carotenoid concentration was calculated according to Equation 3.

(3) $$Carotenoids\;\left(mg\;L^{-1}\right)=7.6[\left(A_{480}-A_{750}\right)\;-1.49(A_{510}-A_{750})]$$

where A represents the absorbance measured at wavelengths of 480, 510, 630, 664, and 750 nm.

### 2.6 Fucoxanthin extraction and HPLC analysis

Fucoxanthin was extracted from 15 mg freeze-dried biomass using 10 mL ethanol. Extraction was performed for 15 min in the dark. The mixture was centrifuged at approximately 9,400 × *g* for 10 min, and the supernatant was collected. Extraction was repeated twice. The combined supernatants were evaporated under nitrogen, and the dry residue was dissolved in 1 mL ethanol for HPLC analysis.

Fucoxanthin was quantified using an Agilent HPLC system equipped with a UV-Vis detector. Separation was performed on a reversed-phase Acclaim 120 C18 column (150 × 4.6 mm, 5 μm; Thermo Scientific) maintained at 30 °C. The mobile phase was water–acetonitrile (1:3, v/v), delivered isocratically at 1.8 mL min^-1^ for 30 min. The injection volume was 10 μL, and detection was performed at 450 nm. Fucoxanthin was quantified using an external standard curve prepared with fucoxanthin standards ranging from 0.5 to 20 mg L^-1^.

Fucoxanthin concentration in culture was calculated using Equation 4.

(4) FC=Fc×DW

where FC is the fucoxanthin concentration in culture (mg L^-1^), Fc is the fucoxanthin content in biomass (mg g^-1^ DW), and DW is the biomass concentration (g L^-1^).

Fucoxanthin productivity was calculated according to Equation 5 as follows:

(5) $$FP=FC_{t}-FC_{0}/t$$

where FP is fucoxanthin productivity (mg L^-1^ d^-1^), FC_t_ is fucoxanthin concentration at time *t, FC_0_ is the* initial fucoxanthin concentration, and t is the cultivation time in days.

### 2.7 FAME analyses

For fatty acid methyl ester (FAME) analysis, 300 mL samples from each treatment were collected after incubation, centrifuged at 8000 × g for 5 min, and freeze-dried using an Alpha 1–2 LD plus freeze dryer (Martin Christ Gefriertrocknungsanlagen GmbH, Osterode am Harz, Germany) for 30 min. The biomass was hydrolyzed and methyl-esterified in 300 μL of 2% (*v/v*) H_2_SO_4_ in methanol at 80 °C for 2 h. Then, 50 μg of C21:0 (heneicosanoic acid; Sigma-Aldrich) was added as an internal recovery standard. After esterification, 300 μL of n-hexane and 0.9% (*w/v*) NaCl were added, the samples were vortexed for 20 s, and then centrifuged at 16,000 × g for 3 min to separate the phases. Next, 150 μL of the hexane phase was analyzed using an Agilent 6890 gas chromatograph (Agilent 6890; Agilent Technologies, Santa Clara, CA, USA) coupled with a 5975 MSD mass spectrometer (Agilent Technologies, Santa Clara, CA, USA), following Agilent’s RTL DBWax method (5988-5871EN) and the protocol of Lim et al. (2012). Fatty acids were identified by comparing retention times with those of external standards (37 FAME Mix, Supelco, Bellefonte, PA, USA) and by reference to the NIST library. Fatty acid components were quantified using the area normalization method, and total fatty acid (TFA) content was calculated as the sum of all identified fatty acids.

### 2.8 Statistical analysis

Mean values and standard deviations were calculated for all treatments. One-way repeated-measures ANOVA followed by pairwise comparisons was performed using SPSS Statistics (version 23) to determine significant differences (*p*< 0.05) between treatments for growth, carotenoid content, and fucoxanthin content. One-way ANOVA with Duncan’s *post hoc* test was used to assess significant differences (*p*< 0.05) between treatments for the fatty acid profile.

## 3 Results

### 3.1 Growth and biomass concentration of *Nitzschia* sp.

Nitrogen availability strongly affected the growth of *Nitzschia* sp. Cells grown without NaNO_3_ reached their maximum density on day 3 and then declined continuously until the end of cultivation. By day 12, the nitrogen-starved culture had decreased to 0.7 × 10^6^ cells mL^-1^, corresponding to a 40.2% reduction relative to the initial cell density (**Figure 1A**).

**Figure 1:**
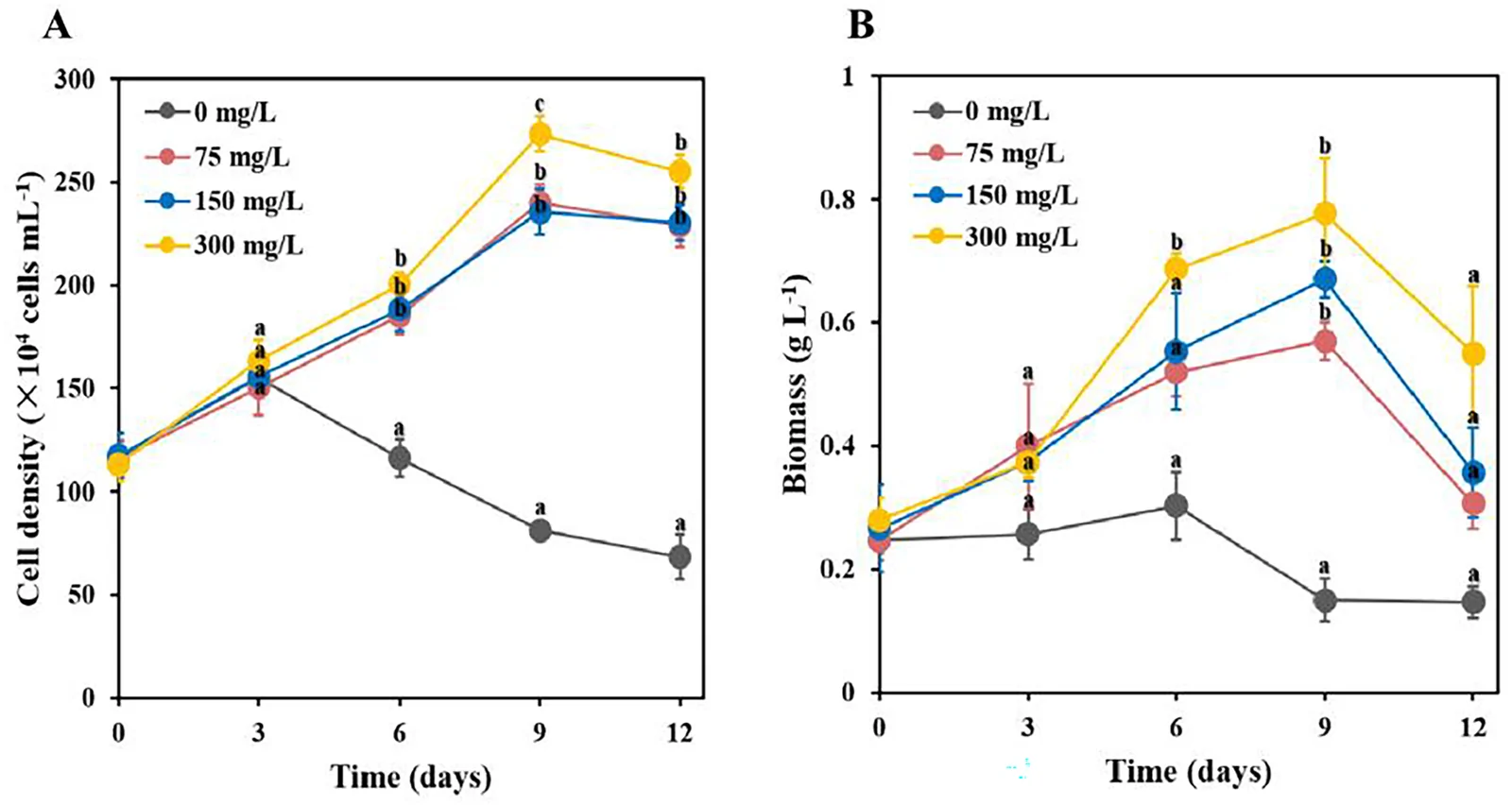
Cell growth and biomass production of *Nitzschia* sp. under different NaNO_3_ concentrations. **(A)** Cell density; **(B)** biomass concentration. Values are expressed as mean ± SD, n = 3. Different letters indicate significant differences among treatments at the same sampling time (p< 0.05).

In contrast, cultures supplied with NaNO_3_ continued to grow until day 9. The highest cell density was recorded under 300 mg L^-1^ NaNO_3_, reaching 2.7 × 10^6^ cells mL^-1^. This value was 2.37-fold the initial density and was significantly higher than that of the nitrogen-starved (*p*< 0.05). However, no significant difference was detected among the 75 and150 mg L^-1^ NaNO_3_ treatments at the peak growth stage (*p* > 0.05).

Biomass concentration exhibited a similar trend. Nitrogen-starved cultures reached a low maximum on day 6 and then declined. The biomass concentration under 0 mg L^-1^ NaNO_3_ decreased by approximately 40% by day 12 compared to the initial value. In nitrate-supplied cultures, biomass generally peaked on day 9. The highest biomass concentration, 0.78 ± 0.03 g L^-1^, was obtained under 300 mg L^-1^ NaNO_3_ (**Figure 1B**).

### 3.2 Pigment accumulation in *Nitzschia* sp.

Total carotenoid accumulation was affected by nitrate concentration. Under 0 mg L^-1^ NaNO_3_, carotenoid concentration declined continuously during cultivation and was 57.5% lower than the initial value by day 12 (**Figure 2A**). In nitrate-supplied cultures, carotenoid concentration increased during the first nine days. The highest carotenoid concentration was obtained under 150 mg L^-1^ NaNO_3_ on day 9, reaching 2.08 mg L^-1^. This value was significantly higher than that under the nitrogen-starved culture (*p*< 0.05).

**Figure 2:**
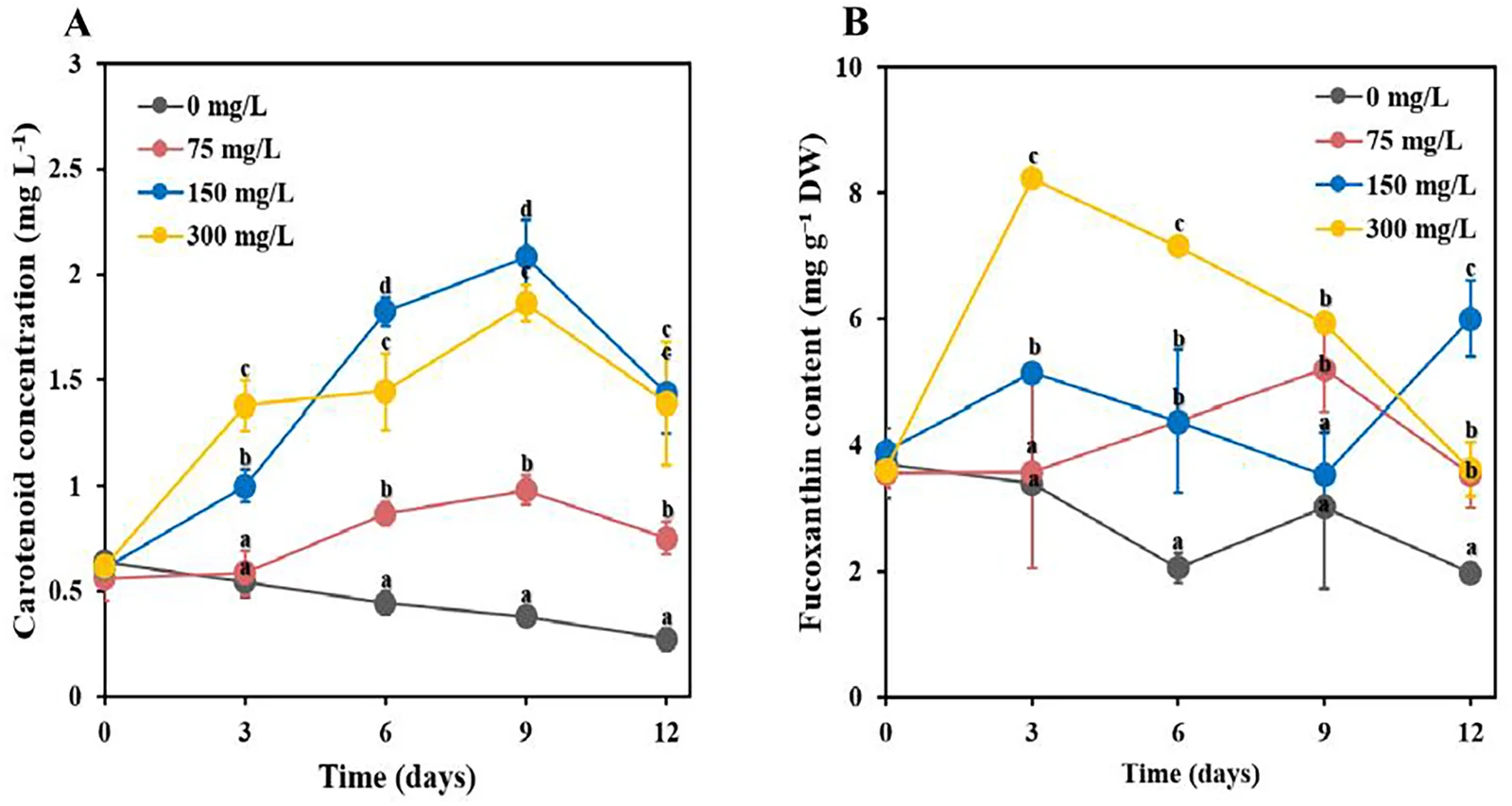
Accumulation of carotenoids and fucoxanthin in *Nitzschia* sp. under different NaNO_3_ concentrations. **(A)** Total carotenoid concentration; **(B)** fucoxanthin content. Values are expressed as mean ± SD, n = 3. Different letters indicate significant differences among treatments at the same sampling time (*p<* 0.05).

Fucoxanthin content also varied with nitrate concentration. Nitrogen-starved cultures maintained low fucoxanthin content throughout the experiment, reaching the lowest value of 1.9 mg g^-1^. The highest fucoxanthin content, 8.42 mg g^-1^ DW, was obtained under 300 mg L^-1^ NaNO_3_ on day 3 (**Figure 2B**). This value was 2.28-fold the initial fucoxanthin content and significantly higher than those under the other treatments. Although fucoxanthin content under 300 mg L^-1^ NaNO_3_ declined after day 3, it remained higher than that in the 0, 75, and 150 mg L^-1^ treatments on days 6 and 9. By day 12, the 150 mg L^-1^ NaNO_3_ treatment showed the highest fucoxanthin content among the treatments, reaching 6.10 mg g^-1^ DW, suggesting that moderate nitrate supply supported fucoxanthin retention during the later stage of cultivation.

### 3.3 Biomass and fucoxanthin productivity of *Nitzschia* sp.

Biomass and fucoxanthin productivities were calculated from changes in biomass concentration and culture-volume fucoxanthin concentration, respectively. Biomass productivity was highest under 300 mg L^-1^ NaNO_3_, reaching 0.068 ± 0.027 g L^-1^ d^-1^ on day 6. Fucoxanthin productivity was also highest under 300 mg L^-1^ NaNO_3_, reaching 0.689 ± 0.175 mg L^-1^ d^-1^ on day 3. Thereafter, fucoxanthin productivity under 300 mg L^-1^ NaNO_3_ decreased to 0.651 ± 0.111, 0.400 ± 0.101, and 0.081 ± 0.037 mg L^-1^ d^-1^ on days 6, 9, and 12, respectively (**Table 1**).

**Table 1:** Biomass and fucoxanthin productivity of *Nitzschia* sp. under different NaNO_3_ concentrations.

Day	NaNO3 (mg L^-1^)	Biomass productivity (g L^-1^ d^-1^)	Fucoxanthin productivity (mg L^-1^ d^-1^)
3	0	0.003 ± 0.022	-0.013 ± 0.077
6	0	0.009 ± 0.014	-0.048 ± 0.033
9	0	-0.011 ± 0.006	-0.054 ± 0.020
12	0	-0.008 ± 0.005	-0.051 ± 0.014
3	75	0.051 ± 0.027	0.153 ± 0.089
6	75	0.046 ± 0.007	0.234 ± 0.036
9	75	0.036 ± 0.004	0.231 ± 0.036
12	75	0.005 ± 0.003	0.017 ± 0.016
3	150	0.036 ± 0.030	0.294 ± 0.124
6	150	0.048 ± 0.013	0.219 ± 0.080
9	150	0.045 ± 0.010	0.146 ± 0.066
12	150	0.007 ± 0.004	0.094 ± 0.045
3	300	0.031 ± 0.049	0.689 ± 0.175
6	300	0.068 ± 0.027	0.651 ± 0.111
9	300	0.055 ± 0.024	0.400 ± 0.101
12	300	0.022 ± 0.016	0.081 ± 0.037

Values are expressed as mean ± SD, n = 3.

### 3.4 Fatty acid composition and EPA/DHA yield

A total of 20 fatty acids were detected in *Nitzschia* sp. after 12 days of cultivation. These included 10 saturated fatty acids (SFAs), 3 monounsaturated fatty acids (MUFAs), and 7 polyunsaturated fatty acids (PUFAs). The dominant fatty acids were C16:0, C16:1n7, C20:5n3 EPA, C22:6n3 DHA, C14:0, and C24:0.

The total identified fatty acid content was lowest under nitrogen-starved and increased with nitrate supply up to 150 mg L^-1^ NaNO_3_. The highest total identified fatty acid content was obtained under 150 mg L^-1^ NaNO_3_, reaching 179.39 mg g^-1^ DW. At 300 mg L^-1^ NaNO_3_, total identified fatty acid content decreased to 121.46 mg g^-1^ DW.

Across treatments, SFAs accounted for 44.67–49.77% of total identified fatty acids, MUFAs accounted for 31.55–35.09%, and PUFAs accounted for 15.14–23.78%. The highest PUFA percentage (23.78%) was observed under 300 mg L^-1^ NaNO_3_ (**Table 2**). However, the biomass-specific yields of EPA (C20:5n3) and DHA (C22:6n3) varied significantly among the NaNO_3_ treatments. EPA yield increased from 3.632 mg g^-1^ DW under nitrogen-starved medium (0 mg L^-1^ NaNO_3_) to 11.301, 25.122, and 20.914 mg g^-1^ DW at 75, 150, and 300 mg L^-1^ NaNO_3_, respectively. Similarly, DHA yield increased from 1.682 mg g^-1^ DW at 0 mg L^-1^ NaNO_3_ to 2.463, 3.517, and 2.932 mg g^-1^ DW at 75, 150, and 300 mg L^-1^ NaNO_3_, respectively. Therefore, the highest EPA and DHA yields were observed at 150 mg L^-1^ NaNO_3_ (**Figures 3A, B**).

**Table 2:** Fatty acid composition and content of *Nitzschia* sp. cultivated under different NaNO_3_ concentrations for 12 days.

Fatty acid	0 mg L^-1^	75 mg L^-1^	150 mg L^-1^	300 mg L^-1^
C4:0	168.68 ± 0.29^d^	98.21 ± 0.60^c^	68.61 ± 0.28^b^	67.21 ± 0.76^a^
C8:0	447.91 ± 0.76^d^	82.43 ± 1.37^c^	63.08 ± 1.42^a^	68.62 ± 0.31^b^
C10:0	351.06 ± 0.74^b^	1214.93 ± 0.56^d^	649.92 ± 1.03^c^	92.03 ± 0.93^a^
C14:0	1066.99 ± 0.35^a^	2421.4 ± 0.36^c^	3401.64 ± 0.36^d^	2230.87 ± 0.09^b^
C15:0	506.34 ± 0.51^a^	623.91 ± 1.07^b^	1093.83 ± 0.47^d^	696.7 ± 0.58^c^
C16:0	12144.25 ± 0.27^a^	47823.68 ± 0.20^b^	73331.12 ± 0.66^d^	47935.42 ± 0.27^c^
C16:1n7	13896.65 ± 0.36^a^	40823.78 ± 0.63^c^	60676.24 ± 0.31^d^	37882.52 ± 0.76^b^
C17:0	1578.19 ± 2.00^d^	660.64 ± 2.06^c^	549.96 ± 1.07^b^	455.79 ± 2.22^a^
C18:0	473.68 ± 1.20^a^	1041.49 ± 1.67^b^	1050.24 ± 1.15^c^	754.87 ± 0.29^c^
C18:1n9c	242.33 ± 2.01^a^	487.15 ± 0.35^b^	631.07 ± 0.45^c^	402.9 ± 0.25^d^
C18:2n6c	613.51 ± 0.32^d^	290.212 ± 2.0^b^	419.59 ± 0.95^c^	280.71 ± 2.10^a^
C18:3n6	286.13 ± 0.96^b^	303.24 ± 1.12^c^	247.61 ± 0.32^a^	423.04 ± 1.95^d^
C20:3n6	92.22 ± 0.75^a^	538.95 ± .025^d^	283.53 ± 0.80^b^	325.08 ± 1.05^c^
C20:4n6	1852.97 ± 0.18^a^	1962.75 ± 0.19^b^	3215.76 ± 0.06^d^	2273.6 ± 0.16^c^
C20:5n3	3632.34 ± 0.36^a^	11301.14 ± 1.01^b^	25121.63 ± 0.32^d^	20914.19 ± 2.04^c^
C22:2n6	1680.03 ± 0.27^b^	993.33 ± 0.90^a^	1993.8 ± 0.35^d^	1729.19 ± 0.76^c^
C22:6n3	1681.84 ± 0.26^a^	2462.84 ± 2.14^b^	3516.6 ± 0.05^d^	2931.74 ± 0.42^c^
C23:0	585.44 ± 0.42^c^	396.59 ± 2.30^b^	614.95 ± 1.80^d^	379.34 ± 0.40^a^
C24:0	3587.80 ± 0.26^c^	4322.95 ± 0.45^d^	2325.87 ± 0.30^b^	1575.21 ± 1.02^a^
C24:1n9	71.14 ± 0.32^c^	60.97 ± 0.301^b^	132.85 ± 1.04^d^	41.85 ± 0.33^a^
SFA	20910.34	58686.23	83149.22	54256.06
SFA (%)	46.51	49.77	46.35	44.67
UFA	24049.16	59224.36	96238.68	67204.82
UFA (%)	53.49	50.23	53.65	55.33
MUFA	14210.12	41371.90	61440.16	38327.3
MUFA (%)	31.61	35.09	34.25	31.55
PUFA	9839.04	17852.46	34798.52	28877.55
PUFA (%)	21.88	15.14	19.40	23.78
FA	44959.50	117910.59	179387.90	121460.88

Values are expressed as μg g^-1^ DW and presented as mean ± SD, n = 3. Values within the same row sharing the same superscript letter are not significantly different (*p* > 0.05).

**Figure 3:**
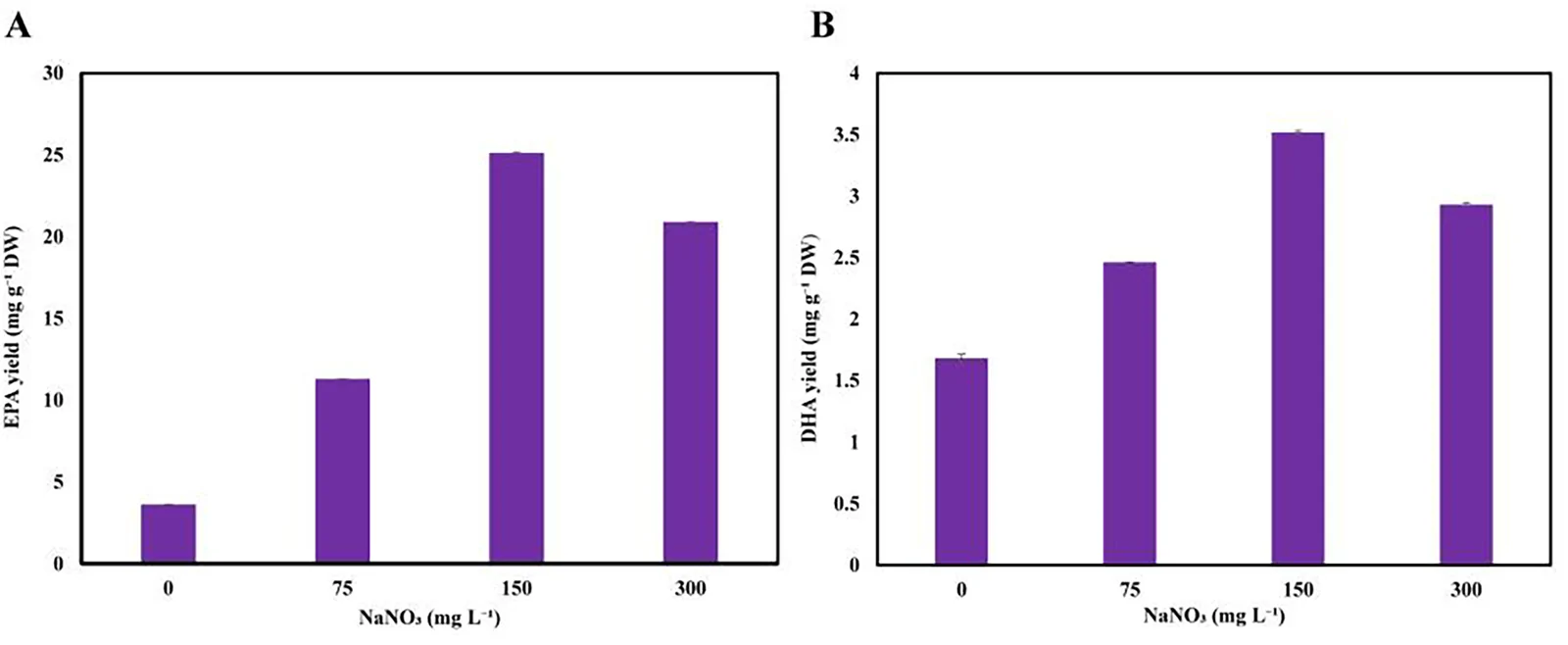
EPA and DHA yield in *Nitzschia* sp. after 12 days of culture. **(A)** EPA yield; **(B)** DHA yield (mg g^-1^ DW). Values are mean ± SD, n = 3.

## 4 Discussion

The present study shows that nitrate supply significantly regulates growth, pigment accumulation, and fatty acid composition in the marine diatom *Nitzschia* sp. The decline of both cell density and biomass under nitrogen starvation, in contrast to the continued proliferation of all nitrate-supplied cultures until day 9, suggest that this isolate has a relatively high nitrogen demand and that nitrate concentrations exceeding those of standard f/2 medium tended to promote biomass accumulation, although cell density did not differ significantly among the 75, 150, and 300 mg L^-1^ treatments at peak growth. ​This result is consistent with studies showing that nitrogen supply can substantially regulate biomass formation and pigment accumulation in *Nitzschia* spp (Sahin et al., 2019; Cao et al., 2022; Grubišić et al., 2024) however, the nitrogen concentration that maximizes growth varies among isolates and cultivation conditions. For example, in alkaliphilic *Nitzschia* sp. NW129, biomass and fucoxanthin productivity increased across a broad nitrogen gradient, with the highest biomass concentration and concurrent maximum fucoxanthin and lipid productivities reported at 117.65 mM nitrogen. Further nitrogen enrichment did not improve biomass and was associated with slight growth suppression, demonstrating that excessive nitrate may become inhibitory rather than beneficial (Cao et al., 2022). Nitrogen optima are therefore strongly dependent on strain identity and culture conditions, possibly reflecting differences in nitrate uptake capacity, intracellular nitrogen storage, growth rate, and adaptation to salinity and pH. A similar reduction in growth under nitrogen-starved conditions was reported for the diatom *Conticribra weissflogii* (Rui et al., 2023).

The pigment response of the present *Nitzschia* sp. also shows both similarities and differences compared with other *Nitzschia* cultures. In this study, nitrogen-starved caused a marked decline in total carotenoids and fucoxanthin, while nitrate enrichment promoted pigment accumulation. Studies of marine *Nitzschia* cultures have similarly shown that nitrogen availability affects fucoxanthin accumulation, although the concentration producing the highest pigment content is not identical among cultures (Sahin et al., 2019; Cao et al., 2022). *Nitzschia* sp. NW129 has been reported as a promising fucoxanthin-producing culture under optimized nitrogen conditions, while *Nitzschia laevis* has also been investigated for fucoxanthin production and associated metabolic pathways (Cao et al., 2022; Lu et al., 2023). Compared with these reports, the present results further demonstrate that fucoxanthin accumulation in *Nitzschia* is strongly dependent on both nitrate concentration and cultivation stage. In particular, the 300 mg L^-1^ NaNO_33_ promoted high early fucoxanthin content and productivity, whereas the 150 mg L^-1^ NaNO_3_ supported greater pigment retention at the end of cultivation.

Higher fucoxanthin accumulation under nitrate enrichment may reflect maintenance of the fucoxanthin–chlorophyll protein antenna system during active growth. This interpretation is consistent with the findings of Xia et al. (2013), who reported that nitrogen-replete cultivation enhanced fucoxanthin biosynthesis in the marine diatom *Odontella aurita*. Consistently, fucoxanthin declined under nitrogen limitation in *Phaeodactylum tricornutum* (Gao et al., 2017)), supporting the view that nitrogen sufficiency helps sustain fucoxanthin synthesis in diatoms.

However, the present results should be interpreted as additional strain- or culture-specific data rather than as evidence of a universal nitrogen optimum for the genus because *Nitzschia* cultures differ in taxonomic identity, light intensity, salinity, temperature, nitrate concentration, cultivation time, and extraction procedure.

The fatty acid response of the present *Nitzschia* sp. was also broadly comparable to the patterns reported for other *Nitzschia* cultures, but important differences were evident. The fatty-acid profile in the present study was dominated by C16:0 and C16:1n7, with EPA as the major long-chain PUFA and DHA present at lower levels. Marine *Nitzschia* cultures examined by Sahin et al. (2019) also showed substantial variation in lipid content and fatty-acid composition in response to nitrogen and iron availability. However, the present culture did not produce its highest identified fatty-acid content under nitrogen-starved condition. Instead, the maximum value occurred under 150 mg L^-1^ NaNO_3_. This result differs from the response often reported for cultures in which severe nitrogen limitation increases the relative abundance of storage lipids. It suggests that, in the present *Nitzschia* sp., moderate nitrate availability was more favorable for the accumulation of the fatty acids detected by FAME analysis than complete nitrogen depletion.

The response of EPA and DHA in the present culture is particularly noteworthy. Both fatty acids reached their highest yield under 150 mg L^-1^ NaNO_3_, and decreased at 300 mg L^-1^ NaNO_3_, despite the higher biomass production. Thus, the nitrate concentration optimizing biomass production did not coincide with that maximizing EPA and DHA yield. Nitrogen-dependent shifts in fatty acid composition have likewise been reported for other *Nitzschia* cultures, with the direction of the response varying among strains and culture conditions (Sahin et al., 2019; Cao et al., 2022). Because both the optimal nitrate supply and the product profile depend on the strain, achieving a given cultivation goal requires appropriate strain selection: *Nitzschia laevis* has been examined mainly for the metabolic regulation and improvement of fucoxanthin productivity (Lu et al., 2023), and *Nitzschia* sp. NW129 for the integrated production of fucoxanthin and lipids (Cao et al., 2022). In the present *Nitzschia* sp., the optimal condition depended on the target product,300 mg L^-1^ NaNO_3_ favored biomass and early fucoxanthin productivity, while 150 mg L^-1^ NaNO_3_ favored total identified fatty acids and EPA/DHA accumulation. This difference reinforces the need to optimize nitrogen conditions separately for each *Nitzschia* culture and for each intended bio-product.

The present results also highlight the importance of distinguishing between relative fatty acid composition (% of total identified fatty acids) and absolute yield (mg g^-1^ DW). In the present study, the highest relative PUFA proportion was observed under 300 mg L^-1^ NaNO_3_, whereas the highest EPA and DHA yields occurred under 150 mg L^-1^ NaNO_3_. Therefore, a higher PUFA percentage did not necessarily correspond to a higher yield of the target fatty acids (EPA and DHA).

The productivity results further show that nitrate-rich conditions are favorable when the production target is fucoxanthin per culture volume. The highest fucoxanthin productivity, was achieved under 300 mg L^-1^ NaNO_3_ (**Table 1**). This reflects the combined effect of high fucoxanthin content and strong biomass accumulation during early cultivation.

Productivity is a key criterion for selecting microalgal strains for the large-scale production of high-value compounds (Mujeeb et al., 2019). Accordingly, nitrogen availability should be optimized with respect to both biomass production and cellular fucoxanthin content. In many fucoxanthin-producing microalgae, nitrogen-replete or elevated-nitrogen conditions promote biomass growth and photosynthetic-pigment accumulation, thereby improving fucoxanthin productivity (Xia et al., 2013; Truong et al., 2024), however, the optimal nitrogen regime is species-specific depends on the cultivation mode (batch and fed-batch) and conditions (Sun et al., 2019).

The present study has several limitations. First, strain identification was based solely on morphological characteristics; molecular confirmation was not performed. Future studies should combine morphology with sequence-based identification, ideally using multiple markers such as the 18S rRNA gene (including the V4 region) and plastid *rbcL*, together with phylogenetic comparison to authenticated reference strains. The deposition of an axenic, molecularly characterized culture in a publicly accessible culture collection would further facilitate traceability and reproducibility. Second, NaNO_3_ was the only nitrogen source evaluated. Because diatoms can assimilate nitrate, ammonium, urea, and, in some cases, other organic nitrogen compounds, comparisons among nitrogen sources may reveal distinct effects on growth, pigment production, lipid accumulation, and fatty-acid composition. Third, the experiment was conducted in controlled flask cultures; consequently, the nitrate concentration identified as optimal under these conditions may not remain optimal during scale-up to closed photobioreactors or outdoor cultivation, where irradiance gradients, self-shading, temperature variation, gas transfer, mixing, contamination risk, and nutrient-delivery dynamics can alter cellular metabolism. Fourth, pigment and fatty acid contents were expressed only per culture volume or per unit dry weight; cellular contents (pg fucoxanthin cell^-1^ and pg fatty acid cell^-1^) were not determined, although they would provide a physiological basis for the observed productivity patterns. Future studies should integrate total lipid quantification, lipid class and TAG profiling, targeted or untargeted pigment metabolomics, and transcriptomic analyses to clarify how nitrogen availability regulates carbon partitioning among membrane lipids, storage lipids, and photosynthetic pigments in this strain.

## 5 Conclusions

Nitrogen concentration significantly influenced growth, pigment accumulation, fucoxanthin production, and fatty acid composition in the marine diatom *Nitzschia* sp. Among the tested treatments, 300 mg L^-1^ NaNO_3_ was most favorable for biomass accumulation and fucoxanthin productivity, whereas 150 mg L^-1^ NaNO_3_ promoted the highest total carotenoid concentration and the highest EPA and DHA yields. These findings show that nitrogen supply can be adjusted according to the desired product, with higher nitrate favoring biomass and fucoxanthin, and moderate nitrate favoring EPA/DHA accumulation. Further studies combining molecular identification, lipid class analysis, and optimization of light, silicon, phosphorus, salinity, pH, and temperature are needed to better understand the physiology of this strain and improve its potential for biotechnological applications.

## Data Availability

The original contributions presented in the study are included in the article/supplementary material. Further inquiries can be directed to the corresponding authors.
